# Novel Proteoliposome-Based Vaccine against *E. coli*: A Potential New Tool for the Control of Bovine Mastitis

**DOI:** 10.3390/ani12192533

**Published:** 2022-09-22

**Authors:** John Quiroga, Sonia Vidal, Daniela Siel, Mario Caruffo, Andrea Valdés, Gonzalo Cabrera, Lissette Lapierre, Leonardo Sáenz

**Affiliations:** 1 Faculty of Veterinary Sciences, Universidad de Chile, Santiago 8820808, Chile; 2 Instituto de Farmacología y Morfofisiología, Facultad de Ciencias Veterinarias, Universidad Austral de Chile, Valdivia 5090000, Chile; 3 Escuela de Medicina Veterinaria, Facultad de Ciencias de la Vida, Universidad Andres Bello, Santiago 8370035, Chile; 4 Escuela de Biotecnología, Facultad de Ciencias, Universidad Santo Tomás, Santiago 8370003, Chile; 5 Programa de Biología Celular y Molecular, Instituto de Ciencias Biomédicas, Facultad de Medicina, Universidad de Chile, Santiago 8380000, Chile

**Keywords:** proteoliposomes, mastitis, murine model, *Escherichia coli*

## Abstract

**Simple Summary:**

Mastitis is a highly prevalent disease in dairy cattle, affecting animal welfare and generating economic losses for the dairy industry. Control measures for coliform mastitis are limited, due to the constant exposure of the teat to bacteria and the emergence of antimicrobial-resistant bacteria, making vaccination an important strategy for control of mastitis. However, currently available vaccines show limited efficacy, which could be attributed to inactivation processes that alter the antigenic preservation of the vaccines. The aim of this study was to assess the efficacy of a novel vaccine against mastitis using proteoliposomes obtained from *E. coli* in a murine model of coliform mastitis. We demonstrated that the proteoliposome vaccine was safe, immunogenic and effective against an experimental model of *E. coli* mastitis, decreasing bacterial count and tissue damage. This proteoliposome vaccine is a potential new tool for prevention of mastitis.

**Abstract:**

*Escherichia coli* is an important causative agent of clinical mastitis in cattle. Current available vaccines have shown limited protection. We evaluated the efficacy of a novel vaccine based on bacterial proteoliposomes derived from an *E. coli* field strain. Female BALB/c mice were immunized subcutaneously with two doses of the vaccine, 3 weeks apart. Between days 5 and 8 after the first inoculation, the females were mated. At 5–8 days postpartum, the mice were intramammary challenged with the same *E. coli* strain. Two days after bacterial infection, mice were euthanized, and the mammary glands were examined and removed to evaluate the efficacy and safety of the vaccine as well as the immune response generated by the new formulation. The vaccinated mice showed mild clinical symptoms and a lower mammary bacterial load as compared to non-vaccinated animals. The vaccination induced an increase in levels of IgG, IgG1 and IgG2a against *E. coli* in blood and mammary glands that showed less inflammatory infiltration and tissue damage, as compared to the control group. In summary, the vaccine based on bacterial proteoliposomes is safe, immunogenic, and effective against *E. coli*, constituting a new potential tool for mastitis control.

## 1. Introduction

Mastitis is an inflammation of the mammary gland with multiple etiologies; however, bacterial infection is the most common associated causative agent [1]. Mastitis has been described as the main cause of economic losses in the dairy industry [2,3], as the inflammatory process decreases the production and quality of milk and has a negative impact on animal health and welfare [4,5]. Antibiotics are normally used as a treatment for mastitis [6]. In several instances, however, these drugs do not work properly due to the development of resistant bacterial strains [7].

*Escherichia coli* (*E.coli*) is a Gram-negative bacteria frequently isolated from acute clinical cases of mastitis [1,8]. The bacteria can invade and proliferate in the mammary gland, releasing various virulence factors, including toxins, antiphagocytic capsules and iron-chelating proteins [9,10]. The bacterial infection and excessive amount of local proinflammatory mediators such as TNF-α and IL-6 that are produced in in response to the bacterial lipopolysaccharide, LPS, can ultimately trigger a severe systemic illness leading to animal prostration and death [11,12,13].

*E. coli* pathogenicity is related to pathogen-associated molecular patterns (PAMPs): LPS, flagellin, and CpG-DNA, which are normally detected by host innate immune cells [14,15,16]. During multiplication and lysis, *E. coli* releases LPS, the main virulence factor of Gram-negative bacteria, initiating an inflammatory immune response [17]. Although most of the LPS is detoxified by immune cells at the site of inflammation, severe bacterial infection can increase the permeability of the blood-milk barrier, allowing LPS, proinflammatory cytokines (mainly TNF-α and IL-1β) and toxic metabolites to enter the bloodstream [18,19]. Detrimental health effects associated with LPS and proinflammatory factors (particularly TNF-α) include fever, changes in the number of peripheral leukocytes, activation of macrophages and the complement system, increased vascular permeability and alterations in plasma levels of metabolites, minerals and hormones. The decrease in milk production during coliform mastitis is directly and indirectly associated with the local and systemic effects of LPS [14,15,20].

The growing demand to avoid health problems, improve animal welfare and reduce antimicrobial resistance makes it essential to identify new preventive mastitis control measures. Continuous teat exposure and the ubiquitous nature of these pathogens make the most widely used procedures, such as pre- and post-milking dipping and local pharmacological drying therapy, ineffective in controlling environmental mastitis caused by coliforms [1,21]. For this reason, vaccination of cows has emerged as a key tool in mastitis control.

The aim of mastitis vaccination programs is to increase the capacity of cows to neutralize intramammary infections, prevent new clinical cases and minimize the alterations caused by infections. In addition, vaccination reduces the spread of different pathogens and restricts the use of antibiotics to treat clinical cases of mastitis. Immunization enhances the immune response mediated by cells and antibodies in blood and milk against specific antigenic determinants [12].

Traditional vaccines based on the “whole cell” concept have an intrinsic immunostimulatory capacity. However, traditional cell antigen inactivation techniques, such as formaldehyde treatment, alter the chemical structure of potential antigenic proteins, limiting their processing by professional antigen-presenting cells (APC), restricting antigenic presentation to T cells and impairing the generation of memory cells [22,23,24]. This could partly explain the low efficacy of currently existing vaccines in the prevention of coliform mastitis [25,26,27]. Vaccines based on attenuated pathogens induce a strong cellular and humoral response [28] but have been associated with adverse side effects, some of them severe and even fatal [29]. Considering these limitations, new strategies have emerged for the development of novel vaccines in recent decades, applying safer technologies, such as subunit and fractionated vaccines [30,31]. Among the latter, the formulation of proteoliposomes of bacterial origin has been suggested as a safe and effective biotechnology for the formulation of vaccines [31].

Bacterial proteoliposomes are lipid vesicles made of one or more concentric lipid bilayers surrounding an internal aqueous compartment. Proteoliposomes may contain, inside or interspersed among the phospholipid membrane, various antigenic molecules derived from a specific bacterial strain [32,33]. Considering the structural versatility, electrical charge, lipid fluidity and size, as well as the ability to incorporate various hydrophilic and hydrophobic antigenic molecules, proteoliposomes constitute an efficient antigen delivery system, inducing humoral and cellular immune responses [34,35]. In addition, the presence of PAMPs in proteoliposomes, such as LPS and bacterial DNA, favors the activation of the innate immune response [31]. The immunogenicity and efficacy of vaccine formulations based on proteoliposomes have been demonstrated against *Salmonella Enteritidis* in chickens [36], *Bordetella pertussis* in mice [37] and *Piscirickettsia salmonis* in trout [38]. Therefore, the use of proteoliposomes derived from bacteria emerges as an alternative for the formulation of vaccines for the prevention of bovine mastitis.

In this work, a vaccine formulation based on *E. coli* proteoliposomes showed high immunogenicity and efficacy in a mouse coliform mastitis model, decreasing the bacterial count at the mammary gland and reducing the severity of disease.

## 2. Materials and Methods

### 2.1. Preparation of Bacterial Proteoliposomes

The proteoliposomes were obtained from a field strain of *E. coli* isolated from a clinical case of bovine mastitis (strain RM5870). Bacteria were grown in Brain Heart Infusion broth (AES Laboratories, Uttar Pradesh, India) for 18 h at 35 °C, under orbital agitation. The culture was then centrifuged at 2800× *g* for 20 min at 4 °C, and the supernatant was discarded. The sediment was washed in sterile phosphate-buffered saline (PBS), incubated in lysis buffer (20 mM Imidazol; 20 mM Na_2_HPO_4_; 500 mM NaCl; pH 7.4) and suspended in Zirconia/Silica 0.1 mm beads (BioSpec Products, Bartlesville, OH, USA). Then, the samples were frozen for 2 h at −80 °C, sonicated (Hielscher Ultrasonic Processor UP400S) and centrifuged at 2800× *g* for 20 min at 4 °C. The sediment was discarded, and the supernatant was centrifuged at 17,300× *g* at 4 °C for 40 min. The resultant membrane pellet was suspended in “membrane solubilization buffer” (20 mM Tris-HCl pH 10; 25 mM KCl; 15 mM sodium deoxycholate) and incubated at 17 °C under orbital agitation for 18 h. The suspension was then centrifuged at 2800× *g* at 17 °C for 40 min, reserving the supernatant with the solubilized membranes. To form the proteoliposomes, detergent was removed from the membrane suspension by adding sterile Bio-Beads^®^ (BioRad, Hercules, CA, USA) and incubating at 25 °C under orbital agitation for 90 min. After Bio-Beads^®^ decantation, the proteoliposome suspension was collected under sterile conditions.

### 2.2. Characterization of Bacterial Proteoliposomes

Proteoliposome total protein concentration was determined using the “BCA Protein Assay Kit” (Novagen^®^, Merck Millipore, Burlington, MA, USA) according to manufacturer’s instructions. The optical density of the protein samples was measured with an Epoch spectrophotometer (BioTek^®^, Winooski, VT, USA) at 562 nm. The protein electrophoretic patterns were observed by 15% SDS-PAGE gel stained with Coomassie blue. The content of LPS in the proteoliposomes was determined by Purpald assay (Sigma Aldrich^®^, St. Louis, MO, USA) as previously described [39]. The incorporation of nucleic acids into the proteoliposomes was evaluated by DNA extraction, applying the phenol–chloroform method [40]. Absorbance was determined using an Epoch spectrophotometer (BioTek^®^) at 260–280 nm. In addition, proteoliposome DNA was electrophoretic separated in 0.7% TAE-agarose gels, stained with GelRed^TM^ Nucleic Acid Gel Stain (Biotium^®^, Fremont, CA, USA) and visualized in a UV transilluminator. For evaluation of morphology and size of bacterial proteoliposome, samples were mounted on 300-mesh formvar/carbon-coated grids (Electron Microscopy Sciences, Hatfield, PA, USA), stained with 2% uranyl acetate for 1 min and examined in a Tecnai 12 BioTWIN (Philips, Amsterdam, The Netherlands) transmission electron microscopy (TEM) operated at 80 kV. The size and Zeta (ζ) potential of the proteoliposomes were evaluated using NanoBrook 90Plus Zeta equipment (Brookhaven Instruments Corp., Holtsville, NY, USA) and the software ZetaPlus Particle Sizing Ver. 5.20 (based on the principles of Dynamic Light Scattering, DLS, for particle sizing and distribution) and Zeta Potential Analyzer Ver. 5.57 (based on electrophoretic light scattering, ELS).

### 2.3. Animals and Vaccination

Sixteen female and eight male (12–14 weeks old) Balb/c mice (*Mus musculus*) were used. The females were randomly divided into two groups (*n* = 8). The vaccinated group was immunized subcutaneously, in the interscapular area, with *E. coli* proteoliposomes (50 μg protein) and aluminum hydroxide as adjuvant (800 μg) prepared in 200 μL PBS. The placebo group was inoculated with 800 μg aluminum hydroxide prepared in 200 μL PBS. Vaccinations were performed on days 1 and 21 of the study.

**Figure 1:**
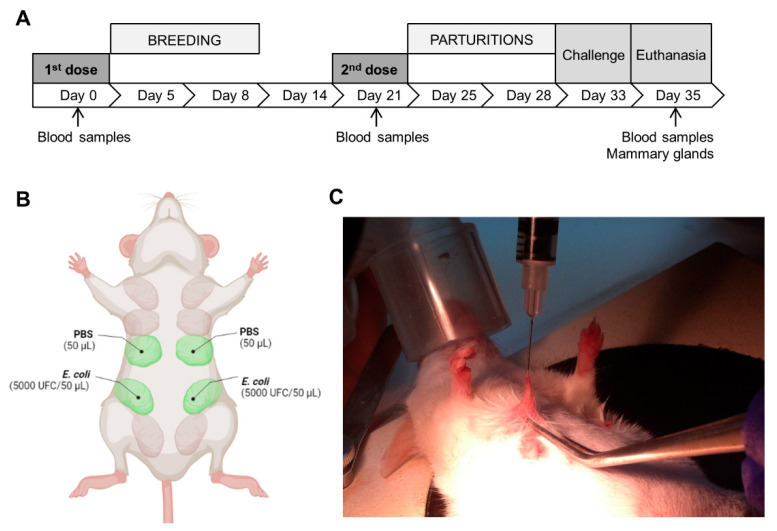
Experimental design and vaccination strategy. (**A**) The first vaccine dose (or placebo) was administered subcutaneously to estrus-synchronized female mice at the beginning of the trial (day 0). Five days after the first immunization, the female mice were mated for 3 days (two female with one male per cage). A second subcutaneous dose of vaccine or placebo was administered on day 21. Intramammary bacterial challenge was performed on day 33 (5 to 8 days postpartum) by inoculating the fourth gland pair with *E. coli* RM5278; the third mammary gland pair was inoculated with sterile PBS as a control (**B**,**C**). At 48 h post bacterial inoculation (day 35), the mice were euthanized, and the mammary glands were extracted for further analysis. Blood samples were obtained on trial days 0, 21 and 35. Figure was created using BioRender.com (accessed on 9 February 2022).

In order to induce pregnancy and lactation, the females were synchronized using male litter, as previously reported [41]. On day 5 of the study, one male and two females were housed in the same cage for mating. After 72 h, the males were removed. Figure 1A shows the summary of the experimental design. Thirty-five days after the first immunization, the animals were euthanized by CO_2_ inhalation.

The animals were maintained under ad libitum feeding conditions, with free access to water, at 24 ± 2 °C with a relative air humidity of about 50 ± 10%. The protocols associated with the maintenance and handling of the animals were approved by the Bioethics Committee, Facultad de Ciencias Veterinarias y Pecuarias, Universidad de Chile (Certificate No. 07-2015).

### 2.4. Intramammary Infection

*E. coli* intramammary infection assays were performed following the protocol described by Chandler et al. [42] with modifications. Briefly, seven days postpartum, pups were removed from the cages. Two hours after this removal, the females were anesthetized with isoflurane, and the fourth pair of mammary glands was inoculated with 5000 colony-forming units (CFU)/gland of *E. coli* strain RM5870, using a 32 G needle through the nipple canal. The third pair of mammary glands was inoculated with the same volume (50 μL) of sterile PBS, as a negative control (Figure 1B,C). Immediately after euthanasia, the mammary glands were aseptically removed and used for immunological, bacteriological and histopathological analysis.

### 2.5. Measurement of Systemic and Local Humoral Immune Responses

To measure serum antibody levels, vaccinated animals were anesthetized using isoflurane, and blood was drawn from the temporal superficial vein on days 1, 21 and 35. The serum levels of IgG, IgG1 and IgG2a against *E. coli* were determined by indirect ELISA assays. Briefly, a 96-well plate (PolySorp™, Nunc™) was incubated with 2 μg *E. coli* RM5278 proteoliposomes in carbonate/bicarbonate buffer (0.15 M Na_2_CO_3_; 0.35 M NaHCO_3_; pH 9.6). Mice sera were tested at 1:1000 (*v*/*v*) dilution and peroxidase-conjugated anti-mouse IgG, IgG1 and IgG2a secondary antibodies were used (1:10,000 *v*/*v*; anti-IgG: donkey, Jackson ImmunoResearch Laboratories Inc., West Grove, PA, USA; anti-IgG1 and anti-IgG2a: goat, Santa Cruz Biotechnology, Dallas, TX, USA). The presence of immune complexes was determined by adding a chromogenic substrate (1-Step™ Ultra TMB-ELISA, Thermo-Fisher Scientific, Waltham, MA, USA). The plates were read using an Epoch spectrophotometer (BioTek) at 450 nm. In addition, for the determination of the antibody profile in the mammary gland, the glands were aseptically extracted and mechanically homogenized in 10 mL/g of sterile PBS. The homogenates were used as primary antibody for indirect ELISA assays against *E. coli*, following the protocol described above, assessing the levels of IgG, IgG1, IgG2a and IgA (1:10,000, donkey, Jackson InmunoResearch Laboratories Inc, West Grove, PA, USA).

### 2.6. Vaccine Effectiveness Evaluation

#### 2.6.1. Clinical Evaluation

To evaluate the capacity of the novel vaccine to prevent the induction of clinical mastitis in the immunized and intramammary infected mice, local mammary and systemic alterations were evaluated three days after the intramammary bacterial challenge, according to the Morton and Griffiths modified protocol [43] (Certificate No. 07-2015). Based on the individual animal clinical score value per day, daily group scores associated with clinical severity were obtained.

#### 2.6.2. Histopathological Evaluation

To evaluate inflammatory and degenerative modifications in the alveolar epithelium, ductal epithelium and glandular stroma, as well as look for the presence of bacteria, mammary histopathological analyses were performed. After the animals were euthanized, the mammary glands were removed and fixed in 10% neutral-buffered formalin. Serial 5 μm mammary gland histological sections were stained with hematoxylin and eosin and evaluated by light microscopy (Olympus FSX100). The total number of cells per visual field was automatically quantified in the histopathological-stained sections using the software ImageJ 1.51n (Dr. Wayne Rasband, National Institutes of Health, Bethesda, MD, USA).

#### 2.6.3. Bacterial Count in the Mammary Gland

To assess the bacterial load in the mammary tissue, the glands were aseptically extracted and mechanically homogenized in 10 mL/g of sterile PBS. Six serial ten-fold dilutions were prepared in sterile PBS and plated in duplicate on McConkey Agar plates and incubated at 35 °C for 18 h. The number of bacteria per plate was counted, and the results were expressed as CFU/g of mammary tissue.

### 2.7. Statistical Analysis

The clinical evaluation as well as the levels of immunoglobulins in blood and mammary glands were analyzed by repeated-measures ANOVA with Tukey’s post-test. Quantitative bacteriological analysis was performed using the Wilcoxon test. For this purpose, the bacterial counts of experimental groups were transformed into logarithmic units (log_10_ CFU/g), with a normal distribution. Data were analyzed using GraphPad Prism program (version 5.01). Differences were considered significant with values of *p* < 0.05.

## 3. Results

### 3.1. Physicochemical Characterization of Proteoliposomes

As shown in Table 1, the prepared bacterial proteoliposomes incorporated potentially immunostimulatory proteins, DNA and LPS. The presence and diversity of bacterial proteins in the formulation was evidenced by SDS-PAGE assays (Supplementary Figure S1).

**Figure 2:**
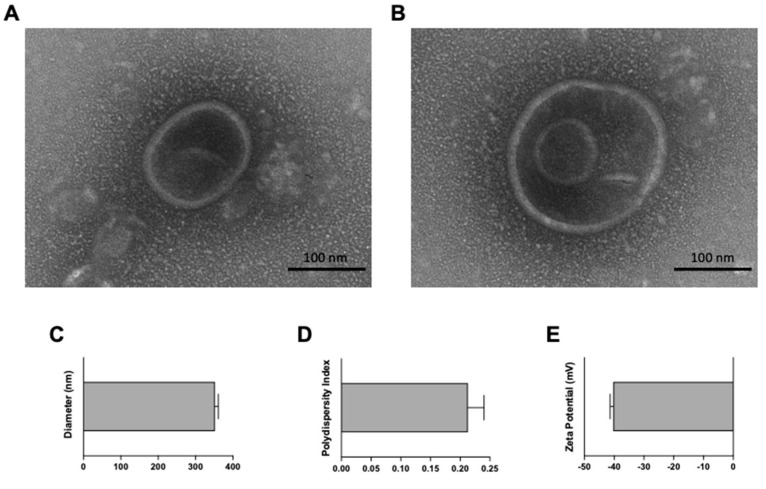
Proteoliposome characterization. Proteoliposome vesicles were observed as monolamellar (**A**) or bilamellar (**B**) rounded vesicles by transmission electron microscopy (TEM) (scale bar = 100 nm). (**C**) Diameter (352.2 ± 8.7 nm), (**D**) polydispersity index (0.213 ± 0.027) and (**E**) Zeta (ζ) potential (−40.33 ± 1.04 mV) were estimated using NanoBrook 90Plus Zeta equipment. Graphs depict the mean ± SD of 10 consecutive measurements.

Proteoliposomes were observed under TEM as round or slightly oval monolamellar (Figure 2A) or bilamellar (Figure 2B) vesicles. The estimated mean size of these vesicles by TEM was 105.517 ± 24.737 nm, with a range from 61.947 to 170.479 nm (Figure 2A,B). However, the average size of the proteoliposomes measured using the DLS technique was 352.2 ± 8.7 nm, significantly larger (*p* < 0.0001) than the mean size determined by TEM (Figure 2C). Additionally, polydispersity and ζ potential were also determined by DLS. Polydispersity, a parameter of the heterogeneity in the distribution of the size [44] showed moderate values (0.213 ± 0.027) (Figure 2D). Finally, the estimation of the ζ potential as an indicator of vesicle surface electrical charge [45] showed negative values (−40.33 ± 1.04 mV) (Figure 2E).

### 3.2. The Novel Vaccine Induces a Systemic Immune Response

**Figure 3:**
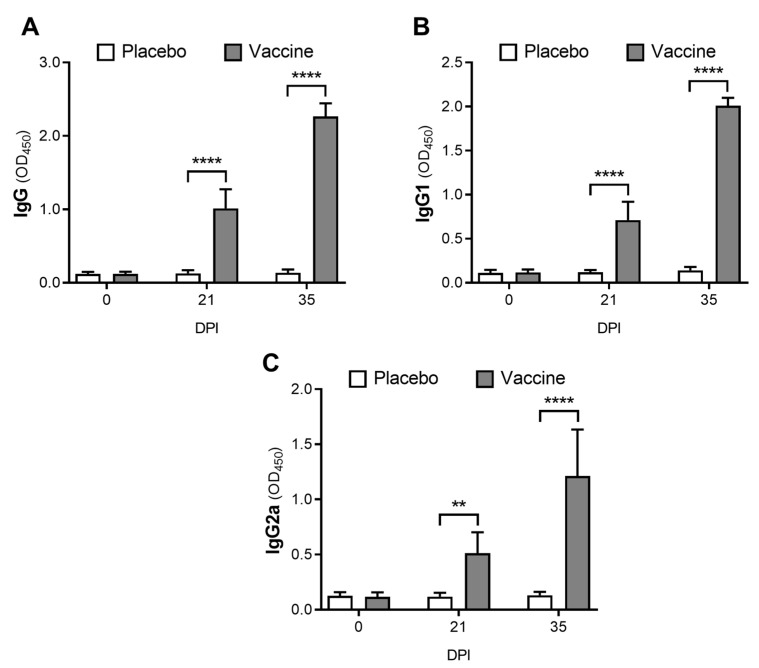
Proteoliposome-based vaccine increases sera levels of IgG, IgG1 and IgG2a against the bacteria. Total IgG (**A**), IgG1 (**B**), and IgG2a (**C**) immunoglobulins against *E. coli* were quantified in the serum of vaccinated and placebo mice, on days 0, 21, and 35, by indirect ELISA assays. Results correspond to means ± SD, n = 8. ** = *p* < 0.01; **** = *p* < 0.0001.

Serum immunoglobulin levels (IgG, IgG1 and IgG2a) against vaccine antigens were evaluated by indirect ELISA assays (Figure 3). IgG levels against vaccine antigens in the immunized group were 7.9-fold higher at day 21 post-immunization (*p* < 0.0001) and 16.6-fold higher at day 35 post-immunization (*p* < 0.0001) than the placebo control group (Figure 3A). Similarly, IgG1 levels were also significantly higher at day 21 (*p* < 0.0001) and at day 35 (*p* < 0.0001) in the vaccinated group (Figure 3B). IgG2a levels were also significantly higher in the vaccinated group at day 21 (*p* = 0.0054) and at day 35 (*p* < 0.0001) than the control mice (Figure 3C).

### 3.3. The Proteoliposome Vaccine Induces a Local Immune Response

**Figure 4:**
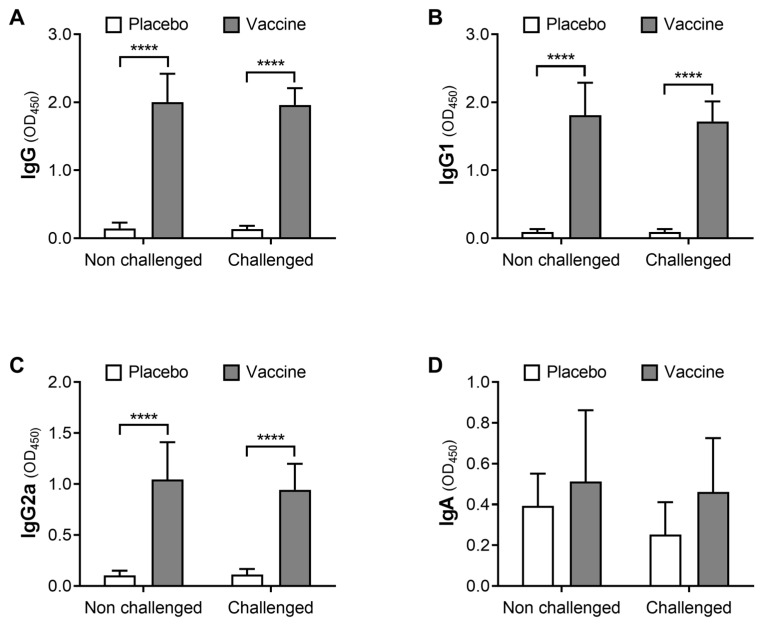
Proteoliposome-based vaccine increases mammary gland local levels of IgG, IgG1 and IgG2a against the bacteria. Total IgG (**A**), IgG1 (**B**), IgG2a (**C**) and IgA (**D**) immunoglobulins against *E. coli* were quantified in the mammary glands of vaccinated and placebo mice on day 35, by indirect ELISA assays. Results correspond to means ± SD, n = 8. **** = *p* < 0.0001.

After euthanasia, the levels of IgG, IgG1, IgG2a and IgA against the vaccine antigens were quantified from prepared mammary gland homogenates (Figure 4). In both intra-mammary bacterial-challenged and unchallenged glands, IgG (Figure 4A), IgG1 (Figure 4B), and IgG2a (Figure 4C) levels were statistically higher in vaccinated mice than in placebo control group mice (*p* < 0.0001). Challenged mammary glands from the vaccinated group showed 14-fold higher levels of IgG (*p* < 0.0001; Figure 4A), 19-fold higher levels of IgG1 (*p* < 0.0001; Figure 4B), and almost 10-fold higher levels of IgG2a (*p* < 0.0001; Figure 4C) than the placebo mice group. Contrarily, the local levels of specific IgA did not show statistical differences between challenged and non-challenged glands (*p* = 0.3415) or between vaccinated and non-vaccinated mice (*p* = 0.1116) (Figure 4D).

### 3.4. Vaccination Protects Mice from Induced E. coli Clinical Mastitis and Reduces the Bacterial Load in Mammary Glands

**Figure 5:**
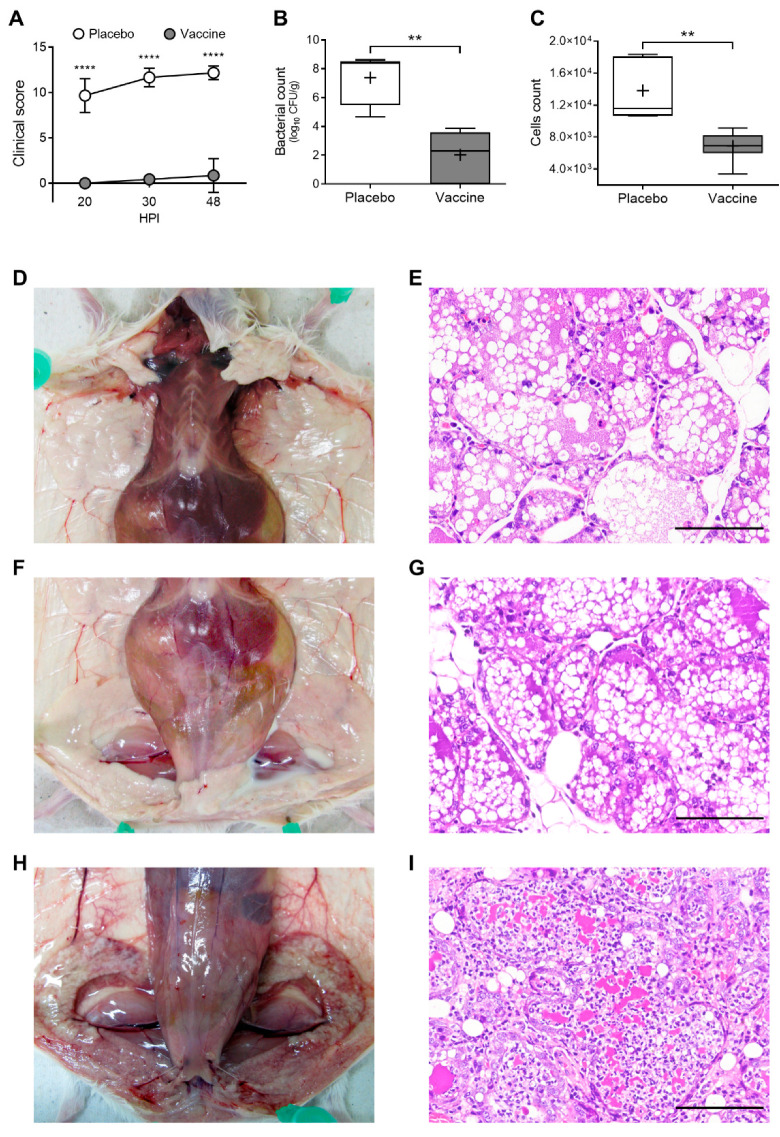
Proteoliposome-based vaccine decreases clinical symptoms, bacterial load and tissue damage after intramammary *E. coli* challenge. (**A**) Clinical score of vaccinated and placebo mice 20, 30 and 48 h after intramammary bacterial challenge (each circle represents the mean ± SD, n = 8). (**B**) Mammary tissue bacterial load of vaccinated and placebo mice, (log_10_ CFU/g). (**C**) Quantification of the total number of cells within mammary glands histopathological sections of vaccinated and placebo mice. Horizontal lines within each box indicate the group median, while the crosses indicate the arithmetic mean (n = 8), ** = *p* < 0.01; **** = *p* < 0.0001. (**D**,**F**,**H**) Macroscopic evaluation of mammary tissue in vaccinated mice (**D**,**F**) and placebo (**H**) at day 35. In vaccinated mice, no evident mammary lesions were observed in glands inoculated with PBS (**D**) and in most of the glands challenged with *E. coli* (**F**). The placebo group shows tissue damage: mammary parenchymal enlargement, edema, congestion, friability, and consolidation (**H**). (**E**,**G**,**I**) Microscopic evaluation of mammary parenchyma in vaccinated mice (**E**,**G**) and placebo (**I**) at day 35. No obvious microscopic changes are observed in the glands inoculated with PBS (**I**) and challenged with *E. coli* in the vaccinated group (**G**); challenged glands from the placebo group show loss of glandular architecture, degraded mammary epithelium, and alveoli filled with massive immune cell infiltration (**I**). Images correspond to representative mice from each experimental group. Stain: hematoxylin and eosin. Bar = 100 µm.

After bacterial challenge, the mice were individually monitored based on a clinical evaluation protocol adapted from Morton and Griffiths [43]. As shown in Figure 5A, the average clinical scores were significantly lower in the vaccinated mice than the placebo control group, at 20 (*p* = 0.0006), 30 (*p* = 0.0006) and 48 (*p* = 0.0012) h post-challenge. In general, the mice in the vaccinated group only showed reddening of the nipples, which was possibly attributed to the trauma caused by needle inoculation. Only two mice showed mild signs of edema and local inflammation 48 h post-challenge. In contrast, all mice in the control placebo group showed moderate to severe mammary swelling 20 h post-challenge, and five mice showed systemic signs of infection. At 30 h post-challenge, all mice in the placebo group had clinical signs such as moderate to severe mammary inflammation, ventral area darkening, moderate to severe depression, elevated respiratory rate and/or asynchronous respiratory rate, wiry coat, kyphotic posture and claudication. The severity of clinical signs and symptoms increased progressively up to 48 h post-challenge. The mice in the placebo group had a significantly higher clinical score 48 h post-challenge than at 20 h (*p* = 0.0253) (Figure 5A).

After euthanasia, quantitative bacteriology analysis was performed using mammary gland homogenates. The bacterial count of the challenged mammary glands was 5.419 log units lower in the vaccinated group (2.018 ± 1.527), than the placebo control group (7.437 ± 1.639), (*p* < 0.001; Figure 5B). Additionally, the total number of cells per visual field was significantly higher in the histopathological mammary gland sections of the placebo group than the vaccinated group (Figure 5C).

### 3.5. Vaccination Decreases Mammary Inflammation and Tissue Damage

Post-mortem analysis of the mice associated the severity of clinical signs and symptoms with the macroscopic alterations of the mammary glands. No mammary lesions were observed in most of the vaccinated mice, and only two mice showed edema and congestion in the challenged glands (Figure 5D). All mice in the placebo group showed macroscopic changes in the mammary tissue, mainly severe congestion and edema (Figure 5E). Surprisingly, four mice from the control group also developed mild edema and congestion in unchallenged glands. However, the histopathological analysis showed healthy and lactating mammary glands in the vaccinated unchallenged glands, with normal lobular and alveolar architecture, intact epithelium, alveoli with exocrine secretion (milk) and few inflammatory cells (Figure 5F). In contrast, the challenged glands of the vaccinated mice presented mild to moderate inflammatory changes, conserving their intact histological architecture, although the alveoli and intralobular ducts presented mild to moderate levels of inflammatory infiltration (Figure 5F). As expected, challenged glands from the placebo control group showed severe inflammatory and degenerative changes, with severe alteration in glandular architecture, degradation of the mammary epithelium, and massive infiltration of inflammatory cells with a predominance of neutrophils (Figure 5G).

## 4. Discussion

Liposomes and nanovesicles derived from liposomes are commonly used as drug delivery systems due to their biocompatibility and biodegradability [46]. The ability of liposomes to induce immune responses against incorporated or membrane-associated antigens has been widely studied, increasing the interest in the development of vaccines based on these structures [47,48]. In 2008, the direct use of bacterial proteoliposomes as antigen carriers was described in the development of a vaccine against *Bordetella pertussis* [37]. Although synthetic liposome formulation is currently described as a highly applicable tool in vaccine development [48], bacterial membrane proteoliposome formulation still presents some technical challenges, particularly in terms of biosafety and reproducibility of vaccine batches.

In this work, we synthesize proteoliposomes from a field strain of *E. coli*, previously isolated from Chilean dairy cows with clinical mastitis. The proteoliposomes were able to retain several bacterial macromolecules with immunostimulatory potential, particularly membrane proteins, DNA and LPS. In SDS-PAGE gels, the presence of quantitatively important bands located between 35–45 kDa suggests the retention of highly antigenic outer membrane porins (Omp) [49,50,51,52,53,54,55,56,57,58]. In addition, highly similar protein electrophoretic patterns in polyacrylamide gels were observed during the production process of *E. coli* proteoliposomes (Supplementary Figure S1), suggesting that proteins retained in proteoliposomes are not denatured during production, contrary to formaldehyde treatment, which alters the chemical structure of proteins, induces protein crosslinking, limiting antigenic processing by APCs and, therefore, restricting antigenic presentation to T cells and the generation of memory cells [22,23,24]. In addition, the retention of bacterial DNA in the formulated proteoliposomes also favors lymphocyte activation through the specific nucleotide binding to Toll-like receptor (TLR) 9 in APCs. Demethylated CpG DNA induces the expression and secretion of IL-6, TNF-α and IL-12, promoting the immune response [59,60].

The LPS content in the formulated proteoliposomes was estimated by Purpald assay as previously described by Lee and Frasch [39]. LPS incorporated into proteoliposomes could stimulate the immune response through the binding and activation of TLR-4 as well as lipopolysaccharide binding protein (LBP), CD14 and MD-2, on APC. LPS induces the expression of proinflammatory cytokines such as IL-1β and TNF-α, co-stimulatory molecules (CD40, CD80 and CD86) and cytokine secretion (IL-12 and IL-2), which increases antigenic presentation to CD4+ T cells, stimulating the adaptive immune response [61].

During the characterization of the *E. coli* proteoliposomes, important differences in the average size of these particles were detected. While the Zeta Plus equipment quantified an average particle size of 352.2 ± 8.7 nm, direct measurement by TEM showed smaller size values (105.517 ± 24.737 nm). For this parameter in particular, direct measurement under a microscope is considered a more accurate approach, since it allows for direct measurement of each proteoliposome, discarding the effect generated by the agglomeration of vesicles. Regardless of these size differences, the polydispersity value provided by the DLS technique (0.213 ± 0.027) suggests that the distribution of the molecular size of the vesicles was heterogeneous. The determination of the size of the proteoliposomes can predict ions of possible recognition by APCs [47,62,63,64]. Ghaffar et al. [46] indicated that small liposomes (20–200 nm) are usually phagocytosed by dendritic cells, while those larger than 500 nm are mainly phagocytosed by cells of the mononuclear phagocyte system and are rapidly removed from the body. The size of the vesicles also influences the profile of the immune response generated. Mann et al. [65] observed that small liposomes (~250 nm) favored Th2-type immune response, while larger ones (~980 nm) induce high levels of IFN-γ and IgG2, typical of Th1-type immune responses.

The ζ potential, an indicator of vesicle surface electrical charge [45,48,66], was estimated to be –40.33 ± 1.03. The maintenance of anionic charges in the proteoliposome formulation depends on the net charge of the bacterial membranes. It has been shown that synthetic proteoliposomes with a net positive or negative charge are more stable compared to uncharged liposomes, since the electrical repulsion forces prevent their agglomeration [45,46,67,68]. Additionally, the internalization of ionized particles by APCs increases up to 1.3 times in relation to neutral ones [69] and the scavenger-type receptors present in APC are able to recognize anionic particles, facilitating their internalization [70].

The protective effect of a vaccine formulated with the proteoliposomes derived from *E. coli* was evaluated in a mouse model of clinical *E. coli* mastitis. Despite the clinical importance of *E. coli* in the etiology of bovine mastitis, most of the preclinical studies using murine mammary glands have been performed with *S. aureus* strains [71,72,73,74,75,76,77,78], although some models of mastitis in mice induced by bacterial LPS are also commonly performed [79,80,81]. In intramammary challenge of 5000 UFC of *E. coli*, we were able to replicate clinical coliform mastitis in all infected mice, generating local and systemic clinical signs. In addition, post-mortem analysis confirmed the presence of macroscopic changes and elevated microbiological counts in mammary glands challenged with *E. coli*.

The *E. coli* proteoliposome-based novel vaccine administered subcutaneously in mice generated a slight volume increase at the inoculation site, which completely subsided within 24 h post-inoculation. All the immunized mice maintained a clinical score of 0 after 72 h post-injection. These results indicate that the immunostimulant components of the novel vaccine were safe and did not generate adverse effects in treated mice, as has been previously demonstrated with other nanovesicle- and liposome-based vaccines [36,37,38,58,82,83,84].

Indirect ELISA assays in blood and mammary gland tissue obtained from inoculated mice showed a significant increase in the levels of antibodies IgG, IgG1 and IgG2a against *E. coli* as early as 21 days post-vaccination, with higher values even at day 35 post vaccination (14 days post-booster). Similarly, the levels of IgG1 and IgG2a were also higher in the mammary gland of immunized mice. Both IgG isotypes are able to fix complement in vivo, and IgG2 is the main opsonin supporting neutrophil phagocytosis in the milk of infected bovine mammary glands [85], since neutrophils and macrophages of cattle have Fc receptors that specifically bind to IgG2 [86]. Furthermore, in both blood and mammary tissue, the high level of IgG1 and IgG2a suggests the development of Th2- and Th1-type responses, respectively. In this regard, the presence of different bacterial molecular patterns in the formulated proteoliposomes may favor Th1-type immune profiles due to the activation of TLRs in dendritic cells [87,88,89,90]. In addition, liposomes have been reported as potent immunostimulants capable of promoting protein antigen-specific IgG production and Th1-associated IgG2a production in mice [91]. Similarly, Reidel et al. [84] evaluated liposomes as adjuvant systems in BALB/c mice using a model protein antigen, resulting in high IgG1 and IgG2a titer production and stimulation of T lymphocytes with IFN-γ production. Furthermore, the levels of specific IgA in the mammary gland were not statistically different between vaccinated and control groups, which was expected considering that mice only induce a local increase in specific IgA after immunization by the same mucosal route [72,76,92].

The administration of the vaccine based on bacterial proteoliposomes was effective in reducing bacterial count and tissue damage in the challenged mammary glands, as well as preventing the occurrence of severe clinical signs and symptoms. Therefore, since the main objective of vaccination is ultimately to decrease the infection incidence and bacterial count at the mammary level, it is highly relevant for the present study that the average bacterial count of the challenged mammary glands in the vaccinated group was 5419 log units lower than the placebo group. These results demonstrate a considerably higher efficacy than other vaccine formulations in experimental murine models of mastitis [71,72,73,74,75,76,77,78]. Furthermore, clinical evaluation of the mice after bacterial challenge also provided valuable information on the efficacy of the vaccine. Clinical scores were significantly lower in vaccinated mice than in mice in the placebo group, which exhibited severe macroscopic mammary changes such as congestion and edema. A reduction in the severity of mastitis is highly beneficial since it can lessen severe potential damage to noble mammary tissue [93]. Comparative histopathological analysis of challenged and unchallenged mammary glands between both experimental and placebo groups showed that the amount of total inflammatory cells in the mammary parenchyma was significantly lower in vaccinated mice than in mice in the placebo group, indicating a lesser degree of inflammatory infiltrate in the mammary gland of immunized mice. Since favorable resolution of intramammary infections by *E. coli* depends on rapid and early recruitment of active neutrophils to the mammary gland [21,94,95,96,97], the lower mammary bacterial count, local tissue damage and clinical score in immunized mice could be explained by the correct functioning of the neutrophil-opsonizing antibody system induced by the proteoliposome formulation. Further research will be needed to characterize the cellular-based immune response against the proteoliposome vaccine and to corroborate its immunogenicity and efficacy in dairy herds.

## 5. Conclusions

Formulated *E. coli* proteoliposomes were formed as lipid vesicles of small size with a net anionic charge that retained various bacterial macromolecules, including proteins, DNA and LPS. Immunization with formulated *E. coli* proteoliposomes in BALB/c mice was highly immunogenic, increasing the levels of IgG, IgG1 and IgG2a against the bacteria in the blood and mammary tissue of immunized mice, suggesting the generation of a Th1 and Th2 immune profile. In addition, the *E. coli* proteoliposome-based vaccine was highly effective in preventing clinical coliform mastitis in a mouse model, showing a strong reduction in mammary bacterial counts and inflammatory cell infiltration in challenged mammary glands compared to a placebo group. Thus, the novel vaccine formulation based on bacterial proteoliposomes was demonstrated to be safe, immunogenic and effective against experimental *E. coli* mastitis, constituting a new potential tool for clinical mastitis control.

## 6. Patents

Chile patent (INAPI) n° 62880 (solicitation number 2014-3247).

## Figures and Tables

**Table 1:** Incorporation of pathogen-associated molecular patterns of bacterial molecules in *E. coli* proteoliposomes.

Molecule	Quantification Result
Proteins (µg/mL)	5596.14 ± 509.05
DNA (ng/mL)	1102.22 ± 114.72
Lipopolysaccharide (LPS) (mg/mL)	97.89 ± 7.54

## Data Availability

Not applicable.

## Supplementary Materials

The following supporting information can be downloaded at: https://www.mdpi.com/article/10.3390/ani12192533/s1. Supplementary Figure S1. Electropherogram of the total protein pattern between different fractions obtained during the production of *E. coli* proteoliposomes. A. SDS-PAGE 12.5% of different fractions obtained from the same proteoliposome production batch (a = bacterial pellet; b = sonicated bacterial pellet; c = bacterial membrane pellet; d = bacterial proteoliposomes). B–F. SDS-PAGE 12.5% analyzed using GelAnalyzer2010a software. Arrowheads of the same color indicate homologous protein bands between the bacterial pellet (C), sonicated bacterial pellet (D), bacterial membrane pellet (E) and bacterial proteoliposomes (F) fractions. *St* = molecular weight standard (PageRuler™ Plus Prestained Protein Ladder, Thermo Scientific); kDa = kilodalton.
