# Supplementary material for: Novel Proteoliposome-Based Vaccine against E. coli: A Potential New Tool for the Control of Bovine Mastitis
Source: Animals (Basel). 2022 Sep 22;12(19):2533. doi: 10.3390/ani12192533 (PMC9558995; doi:10.3390/ani12192533)
Supplement: Supplementary file 1 [file animals-12-02533-s001.zip › animals-1866305-supplementary.pdf]

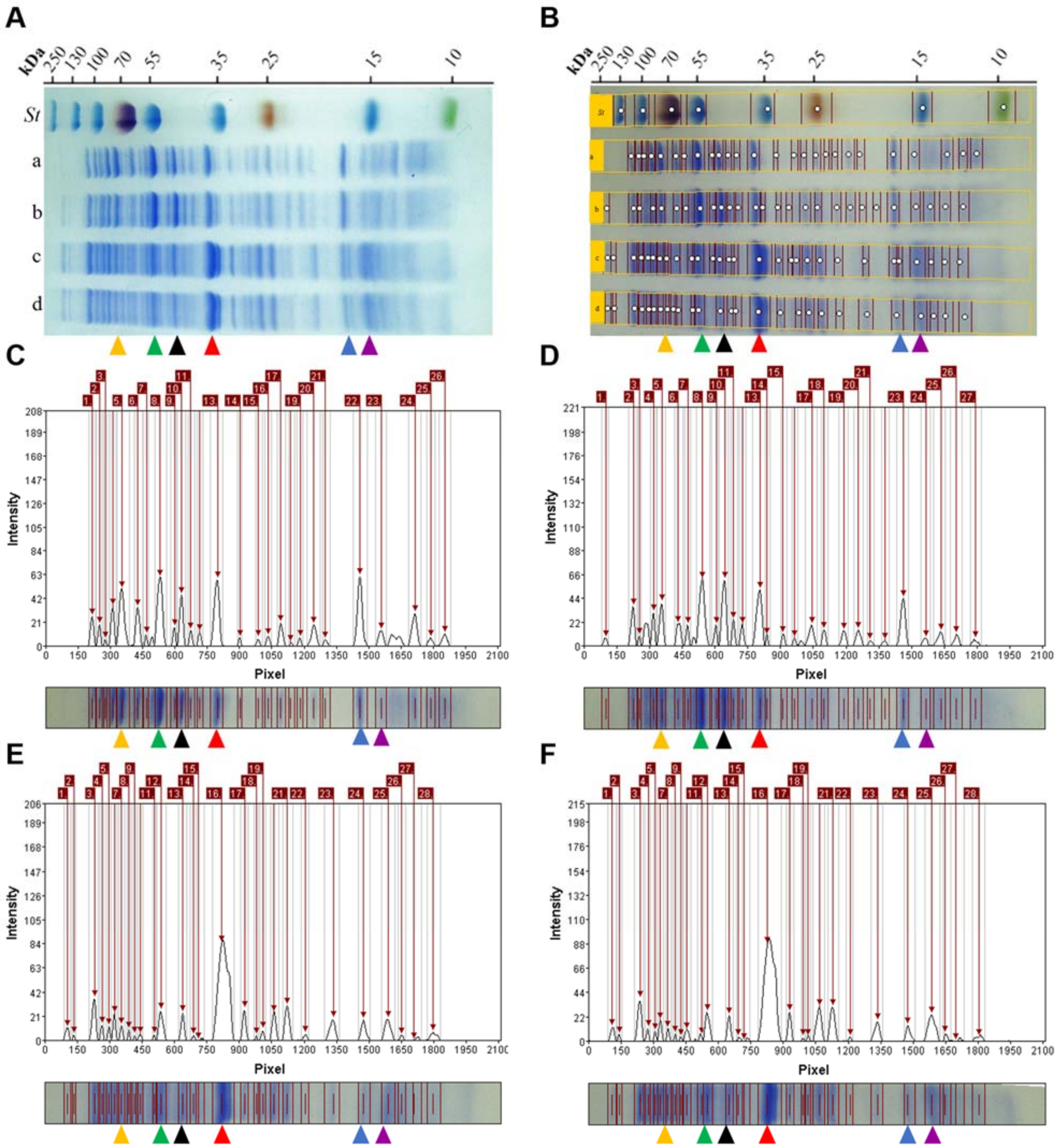

Supplementary Figure S1. Electropherogram of the total protein pattern between different fractions obtained during the production of *E. coli* proteoliposomes. A. SDS-PAGE 12.5% of different fractions obtained from the same proteoliposome production batch (a = bacterial pellet; b = sonicated bacterial pellet; c = bacterial membrane pellet; d = bacterial proteoliposomes). **B-F.** SDS-PAGE 12.5% analyzed using GelAnalyzer2010a software. Arrowheads of the same color indicate homologous protein bands between the bacterial pellet **(C)**, sonicated bacterial pellet **(D)**, bacterial membrane pellet **(E)** and bacterial proteoliposomes **(F)** fractions. *St* = molecular weight standard (PageRuler™ Plus Prestained Protein Ladder, Thermo Scientific); kDa = kilodalton.
